# Pitching Emotions: The Interpersonal Effects of Emotions in Professional Baseball

**DOI:** 10.3389/fpsyg.2016.00178

**Published:** 2016-02-16

**Authors:** Arik Cheshin, Marc W. Heerdink, Jolanda J. Kossakowski, Gerben A. Van Kleef

**Affiliations:** ^1^Human Services, University of HaifaHaifa, Israel; ^2^Psychology, University of AmsterdamAmsterdam, Netherlands

**Keywords:** emotion, interpersonal effects of emotion, social influence of emotion, competitive sports, anger, worry, happiness

## Abstract

Sports games are inherently emotional situations, but surprisingly little is known about the social consequences of these emotions. We examined the interpersonal effects of emotional expressions in professional baseball. Specifically, we investigated whether pitchers’ facial displays influence how pitches are assessed and responded to. Using footage from the Major League Baseball World Series finals, we isolated incidents where the pitcher’s face was visible before a pitch. A pre-study indicated that participants consistently perceived anger, happiness, and worry in pitchers’ facial displays. An independent sample then predicted pitch characteristics and batter responses based on the same perceived emotional displays. Participants expected pitchers perceived as happy to throw more accurate balls, pitchers perceived as angry to throw faster and more difficult balls, and pitchers perceived as worried to throw slower and less accurate balls. Batters were expected to approach (swing) when faced with a pitcher perceived as happy and to avoid (no swing) when faced with a pitcher perceived as worried. Whereas previous research focused on using emotional expressions as information regarding past and current situations, our work suggests that people also use perceived emotional expressions to predict future behavior. Our results attest to the impact perceived emotional expressions can have on professional sports.

“… There was pride in Casey’s bearing and a smile lit Casey’s face.

And when, responding to the cheers, he lightly doffed his hat,

No stranger in the crowd could doubt ‘twas Casey at the bat.

… The sneer is gone from Casey’s lip, his teeth are clenched in hate,

He pounds with cruel violence his bat upon the plate;

And now the pitcher holds the ball, and now he lets it go,

And now the air is shattered by the force of Casey’s blow.

Oh, somewhere in this favored land the sun is shining bright,

The band is playing somewhere, and somewhere hearts are light;

And somewhere men are laughing, and somewhere children shout,

But there is no joy in Mudville – mighty Casey has struck out.”

— (from *Casey at the Bat* – Ernest Lawrence Thayer)

## Introduction

Sports are a natural breeding ground for emotions, and baseball is no exception – as is evident from the famous poem by Ernest Lawrence Thayer. Scientific evidence, too, indicates that even the “masculine” context of professional American baseball is ridden with emotions, which are commonly expressed during matches (MacArthur and Shields, 2015). However, it remains unclear how observers respond to these emotional expressions. Here we report one of the first studies on the interpersonal consequences of emotions in the context of professional sports. Specifically, we investigated whether pitchers’ perceived facial emotional displays influence how their pitches are assessed and responded to.

Emotions are not just private feelings – they tend to be expressed, oftentimes in the presence of others (Parkinson, 1996). This means that other people may perceive emotional expressions and may be influenced by them (Keltner and Haidt, 1999). According to emotions as social information (EASI) theory (Van Kleef, 2009), this influence may come about via two distinct processes. First, emotional expressions may evoke affective reactions in observers, which may in turn influence their behavior. A considerable body of research has documented evidence of various types of affective reactions and their downstream consequences, the most widely studied process being emotional contagion (Hatfield et al., 1993). This refers to the tendency of individuals to (unconsciously) “catch” the emotions of others. The resulting emotional states may in turn influence people’s cognitions, attitudes, and behaviors (Forgas, 1995). Second, emotional expressions may elicit inferential processes in observers (Van Kleef, 2009). According to appraisal theories of emotion, emotions arise in response to events that are perceived as relevant to important concerns or goals (Frijda, 1986; Scherer et al., 2001; Ellsworth and Scherer, 2003). For example, anger tends to arise when one’s goals are frustrated and blame can be ascribed, happiness tends to arise when goals are attained or good progress is being made, and worry tends to arise when there is uncertainty about the future attainment or thwarting of one’s goals. Based on this notion, it is possible to glean information from others’ emotional expressions by a reversal of the appraisal process, such that the emotional expressions of others are used as information about how the expresser interprets the situation (Manstead and Fischer, 2001; Hareli and Hess, 2010; Van Doorn et al., 2012). Thus, expressions of anger may be interpreted as a sign of goal blockage and other blame, expressions of happiness may be taken as a sign of goal achievement, and expressions of worry may be taken as a sign of insecurity about the future.

A growing body of research speaks to the social consequences of emotional expressions (for reviews, see Van Kleef et al., 2011, 2012). In particular, studies across a variety of domains have demonstrated that observers use others’ emotional expressions to gain insight into the expresser’s goals and desires and to inform their understanding of situations. Negotiators use emotional expressions of their counterparts to locate the counterpart’s limits and to determine their own strategy (Van Kleef et al., 2004; Sinaceur and Tiedens, 2006). Individual group members use the emotional expressions of their fellow group members to gage their momentary levels of acceptance in the group and to decide whether they should conform to the majority or have the leeway to deviate (Heerdink et al., 2013). Service employees use the emotional displays of customers to determine the credibility of their complaints (Hareli et al., 2009). Work teams use the emotional displays of their leaders to gage the quality of their performance and to calibrate their effort expenditure (Sy et al., 2005; Van Kleef et al., 2009). Outside observers use the emotional expressions of team members to arrive at inferences regarding the team’s cohesion, cooperation, and conflict (Magee and Tiedens, 2006; Homan et al., 2016). Thus, it is clear that emotions provide social cues for those who notice them.

When it comes to competitive sports, any information that may provide insight into how the opposing team is going to play is greatly sought after. Knowing the tendencies and preferences of specific players on the other team can greatly influence the tactics for the game. For example, in baseball, knowing a batter’s tendency for swinging only at certain pitch types, or a pitcher’s tendency to throw low balls in specific situations, can impact the way that a player prepares for a pitch or swing. This can be evident from the following quote from former professional baseball player and coach Charlie Metro:

The good hitters get their tip-off from the pitchers. And there are many, many ways that a pitcher tips off his pitches. He grips it like that [fingers straight over top of ball]; there’s your fastball. When he throws a curveball, he chokes the ball [wedges it between his thumb and forefinger, gripping it on the side so it sticks out]. Now see how much white of the ball shows on a fastball? And how much more white shows on a curveball?... Another thing is when they bring the ball into the glove, when they come in with a flat wrist like that, that’ll be a fastball. When they turn their wrist like that, it’s a breaking pitch. There are many, many ways, and the good hitters pick out these things... facial expressions... human habits and characteristics will tell. (Carlson and Charlie, 1999, “Biological Baseball”, para 4).

As a result, scouting reports for players are big business in baseball, as in any sports (consider the biographical Hollywood sports drama *Moneyball*).

In this paper we build on the general notion that emotional expressions provide relevant information (Parkinson, 1996; Keltner and Haidt, 1999; Manstead and Fischer, 2001; Van Kleef, 2009) to examine what type of information observers distil from perceived pitchers’ emotional expressions during professional baseball games, and to investigate whether batters are indeed influenced by the pitcher’s facial expressions, as Charlie Metro’s quote suggests.

Although research on the effects of emotional displays in the context of sports is scarce (Friesen et al., 2013), there is some evidence that the emotional displays of players can indeed influence the trajectory of sports games. Totterdell (2000) examined processes of mood convergence and linkage among teammates during professional cricket matches. He found that the moods of players of the same team were more strongly linked than the moods of players of different teams, and that positive moods of players were associated with subjective ratings of performance. Totterdell’s study thus provided evidence for the occurrence of affective reactions to others’ emotional displays in the context of professional sports as well as suggestive evidence that such affective reactions may be associated with team performance. The current study complements this earlier work by focusing on the role of inferential processes as opposed to affective reactions (see Van Kleef, 2009). Furthermore, whereas Totterdell’s seminal study focused on how players’ and teams’ own moods were associated with their (subjective) performance, the current research examines how observers use the perceived emotional displays of sports players (in this case, pitchers in baseball) to make predictions about their actual subsequent, physical performance (i.e., how fast a ball is going to be thrown; how close the ball is going to be thrown to the target, etc.). Moreover, we examine the relationship between the pitcher’s perceived emotional display and the batter’s tendency response to these displays (either to approach the ball and swing or to avoid the ball and not swing).

In the current research we extend the theoretical notion of EASI (Van Kleef, 2009) to the domain of professional sports by asking the following questions: Is it possible that, in the brief few seconds prior to a pitch, the perceived emotional displays of the pitcher could provide information about what is about to occur? Would this information be detected by observers? Would this information be valid and valuable such that it could predict the quality of the throw by the pitcher and consequently the behavior of the batter? The relevance of any answers to these questions goes beyond the baseball context or the sports context more generally. Investigating what information observers’ draw from others’ perceived emotional displays in real-life settings contributes to a more complete understanding of the interpersonal effects of emotions.

This research further extends current knowledge by tying perceived emotional displays to predictions of physical actions and by focusing on predicting future behavior. Specifically, the outcomes we measure are how fast and how accurate a pitcher would throw a ball, and how likely another person would be to attempt to hit a ball that is thrown toward him. To date most research on inferences has been limited to character judgments (e.g., Knutson, 1996; Hareli and Hess, 2010), goals and intentions, (Van Kleef et al., 2004), past performance (e.g., Van Kleef et al., 2009), credibility of complaints (Hareli et al., 2009), and the construal of social situations (e.g., Van Doorn et al., 2012, 2015a). This work has shown that emotions of others serve as cues regarding a situation that has already occurred. None of this previous work has looked at predictions of future behavior, and even more specifically in our case the prediction of physical performance.

### Setting – American Baseball

**FIGURE 1:**
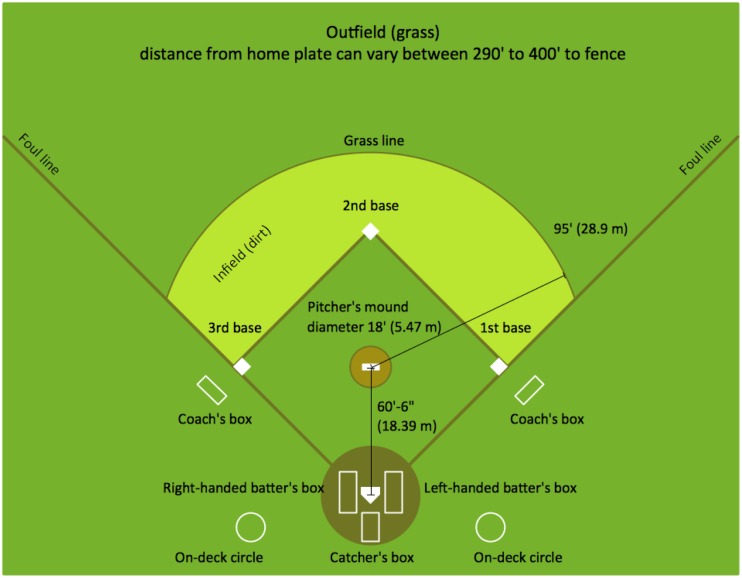
**A diagram of the baseball field – taken from http://www.conceptdraw.com/solution-park/sport-baseball**.

American Baseball is an ideal setting in which to investigate interpersonal effects of emotions in sports, because it involves a game situation wherein two individuals from opposing teams are set one against the other in a form of a duel. This duel has a clear outcome that can take several forms that can be objectively determined. It involves both parties facing each other, within viewing distance, where one initiates, and one responds. Moreover, video footage and records of actual qualities of pitches and reactions to pitches are available, making all of the reactions objectively quantifiable. To facilitate the reader’s understanding of our procedure and analyses, Appendix 1 provides a short description of the game of baseball and **Figure 1** shows a diagram of the baseball field.

## Overview of the Current Research

We tested how observers of pitchers in baseball games make use of pitchers’ facial displays to assess specific aspects of a pitch that is about to be thrown. We also examined the ability of observers to successfully predict the batter’s response to the pitch. Using data from U.S. Major League Baseball games (the “World Series”), we presented short video clips to Dutch students, instructing them to assess various aspects of an upcoming pitch as well as the possible responses of the batter. We later matched the predictions of the students to the actual features of the pitches in those games.

Because it is not possible to survey professional baseball players on the emotions they recognized before they decided to respond to a pitch, we chose what we believe is the best available means for testing our predictions – using TV footage. The TV footage allows us to show the stimuli as they occurred in reality and “pause” that reality for an assessment of the emotion and prediction, and then “un-pause” the footage to test for the real-life result. Thus, our design uses real-life stimuli as well as real-life results taken from baseball games, while we rely on laboratory data and student participant assessments in order to test our predictions.

Although we aimed for the most ecologically valid methods to test our predictions, the design we used does come with imperfections. The real-life setting has the pitcher and the batter facing one another at a distance of about 18 m; while the footage of the pitcher that is viewed by student participants from TV-footage is enlarged due to the ability of the camera to focus and zoom in to enlarge the pitcher’s body and face. This is not equivalent to the reality of the actual match. Moreover, the batter has in his line of sight many other aspects that the students participating in our lab study do not see. The batter sees the rest of the field, his teammates, the opposing team members, the crowed, etc. Our student participants focus solely on the pitcher. In addition, the batters are under pressure during the match which could determine the winners of the championship, and are required to not only assess the emotion but also to prepare themselves with a response. In the lab our student participants are not professional baseball players. They are not trained at assessing emotions, especially not those of pitchers who move as they are about to pitch a ball, nor are they under pressure to perform. To add to that, our sample (which will be described below) was made up of Dutch students, who have limited exposure to baseball. Thus, our design does not actually mirror reality. Yet, we have little doubt that batters do have the ability to view the pitchers’ body and face and to assess their emotion, as is evident from the quote above from professional player and coach Charlie Metro. Players are trained at focusing on the pitcher and acquiring cues regarding the upcoming pitch, which includes assessing emotions of pitchers. Thus, the lack of training and ability of our student participants in decoding the pitcher’s emotional expression should partially compensate for their larger image of the pitcher, relative to the professional players.

We first collected the material – video clips showing facial expressions of pitchers before a pitch. Then the material was assessed for emotional cues – to see if emotions could be detected in a consistent manner. Once that had been established, we examined what kind of information naïve participants extracted from these identified emotions. The final step involved comparing the participants’ assessments to actual game outcomes (i.e., from the World Series) regarding the pitch and the response to the pitch by the batter.

## Data Analysis

**FIGURE 2:**
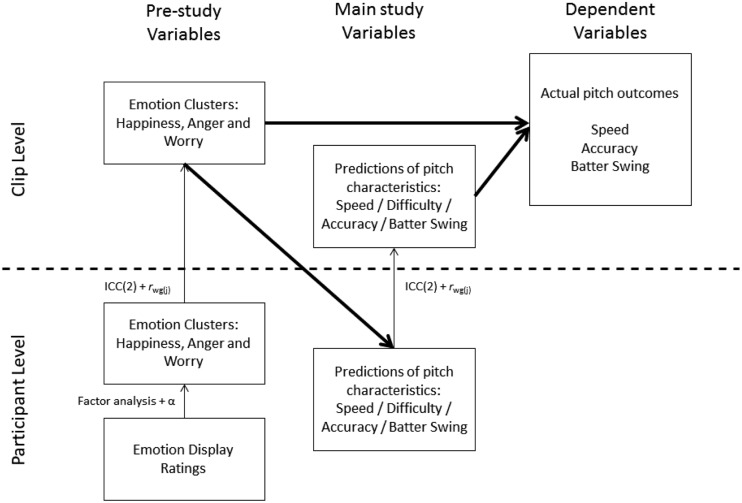
**Schematic representation of the multilevel nature of the data and the data analyses.** Narrow arrows represent aggregation (the indicated analytical techniques were used as criteria before aggregation) and bold arrows represent IV to DV relations. Analyses within one data level (e.g., using only clip-level variables) were conducted using generalized linear models (GLMs), and analyses using variables from both levels were conducted using generalized mixed-effect models (GLMMs). The final mediation analysis is not indicated in this schematic overview for the sake of clarity.

To facilitate the understanding of the multilevel nature of our data and data analyses, we have summarized our analytical approach in **Figure 2**. On the participant level (lower half of **Figure 2**), we collected emotion display ratings (pre-studies) and predictions of pitch characteristics (main study). These ratings were aggregated to the higher clip level (upper half of **Figure 2**) and regressed on the actual outcomes of the pitch (as indicated by archival data from the MLB). Within one level of analysis, we used factor analyses, reliability analyses (assessed with Cronbach’s α), and generalized linear models (GLMs). Some analyses involved multiple levels of analysis, like the assessment of inter-rater reliability and inter-rater agreement before aggregating rating from the participant level to the clip level, and the use of generalized mixed effects models (GLMMs) for analyses at both levels. We will discuss these analyses in turn.

Prior to aggregating participant-level ratings to the clip-level, we assessed both the inter-rater *reliability* and the inter-rater *agreement* of the individual perceived emotion display ratings (as recommended by LeBreton and Senter, 2008). Inter-rater reliability, commonly measured with intra-class correlation measures such as ICC(2) (Shrout and Fleiss, 1979), reflects the extent to which individuals reliably rank-order the clips in terms of the displayed emotions. The reported ICC(2) values were derived from a GLMM (see explanation below) that was fitted using the lme4 package (version 1.1.9; Bates et al., 2015) for R (version 3.2.2; R Core Team, 2015), and bootstrapped using the bootMer function (10,000 resamples). We report the 95% confidence interval for the ICC(2) statistic. Inter-rater agreement, which is commonly determined using the *r*_WG(J)_ index, reflects the extent to which judges are equivalent in terms of the absolute perceived emotion display score they assign to a video clip. The reported *r*_WG(J)_ values may be interpreted as follows: Values below 0.30 reflect a lack of agreement, values between 0.31 and 0.50 reflect weak agreement, values between 0.51 and 0.70 reflect moderate agreement, values between 0.71 and 0.90 reflect strong agreement, and values between 0.91 and 1.00 reflect very strong agreement (LeBreton and Senter, 2008). Because our main interest is in the consequences of *relative* differences in perceived emotion displays between clips, rather than in the absolute degree of perceived emotion displayed in the videos, we deemed satisfactory inter-rater reliability (i.e., ICC[2] > 0.70, Bliese, 2000) more important than inter-rater agreement. We therefore used inter-rater reliability as our primary selection criterion and inter-rater agreement as a secondary criterion.

Whenever our analysis involved both a participant-level outcome and clip-level predictors, we used GLMMs that were fit using the (g)lmer function in the lme4 package (Bates et al., 2015) for R (R Core Team, 2015). An extensive discussion of GLMMs is beyond the scope of this article, but it is useful to note how the inclusion of both fixed and random effects in a model (which is what ‘mixed-effect’ in the name of the technique refers to), benefits our analyses. Fixed effects are identical to the parameters in a regular GLM, and refer to the effect of interest (e.g., the relation between anger displays and estimated pitch speed). The (one or more) *random* effects, however, are unique to mixed-effect models and allow controlling for higher-level covariance while estimating effects on a lower level. Thus, including a random effect for clip while regressing clip-level anger displays on participant-level estimates of pitch speed controls for clip-level variance in estimated pitch speed that is not accounted for by (and unrelated to) the fixed effects (e.g., due to unique clip characteristics such as an exceptionally muscular pitcher) that would otherwise obscure or inflate the estimated relation between anger displays and estimates of pitch speed. The use of a GLMM thus gives us a better focus on the effects of interest. For an introduction to mixed effect linear models and their application to multilevel data, see Field and Wright (2011), and for more extensive coverage of the topic, see West et al. (2014).

## Pre-Study: Selecting and Testing Instances of Perceived Emotional Displays

We conducted a pre-study in which participants were asked to assess the emotional displays of various pitchers based on short video clips. The goal of this study was to test whether observers can reliably identify pitcher’s emotion based on facial displays in these video clips. The study was carried out in accordance with APA regulations and approved by the IRB at the University of Amsterdam.

### Method

#### Participants

Dutch undergraduate psychology students participated in the study and were compensated by pay (€3.50) or partial class credit. The 2011 sample included 151 participants (age *M* = 22.06, range 18–64, 63 men, 81 women, seven missing demographic information) while the 2012 sample included 62 (age *M* = 22.81, range 18–41, 26 men, 36 women).

#### Materials

We selected video clips from high-stakes games that are likely to be among the most emotional of all the season – the final games of the World Series. The clips were selected from the last two games of the 2011 finals (games 6 and 7) and from the final game of the 2012 finals (game 4). These games determined who would win the Major League Baseball (MLB) championship. TV footage of the games was screened for incidents where the face of the pitcher could clearly be seen right before the pitch^1^, as there are many incidents where the camera is focused on the batter or on other elements of the game, and not on the pitcher.^2^ In the 2011 finals (games 6 and 7) there were 659 pitches in total, of which 63 met the above mentioned inclusion criterion. In the 2012 finals there were 290 pitches in total, of which 29 met the inclusion criterion.

Once we identified these incidents, we created short clips that lasted only a few seconds, ranging from 1.5 to 3 s (the material is available open request from the corresponding author). These clips were further edited in order to blur all irrelevant information (e.g., team names, the score, players on base, etc.) so that they showed only the pitchers’ faces and bodies as they prepared to throw the ball.

#### Procedure

The short clips were shown in randomized order to participants. Following the clip, participants were asked to indicate on a scale from *1 = not at all* to *10 = very much* the degree to which a number of emotional displays had been visible in the clip. The list of emotions was prepared by the researchers in advance and contained those emotions that were deemed most relevant given the current context. The exact items differed somewhat between the 2011 and 2012 finals (details below). Participants received a notification before each clip was about to start. There was no possibility to rewind and watch a clip more than once.

### Results

#### Aggregation Strategy

The aggregation from the individual, per-emotion ratings to clip-level perceived emotion displays proceeded as follows. First, we assessed the reliability of the emotion scales using the average of the per-clip reliabilities, instead of calculating an overall reliability across all observations. (The latter approach would treat multiple observations from the same individual as independent, which would inflate the estimated reliability). Then, we determined the agreement among the raters about the extent to which various emotions were displayed in the video clips (see Data Analysis above for more details) prior to aggregating the perceived emotion ratings to the video level.

#### 2011 Finals Clips

The 63 clips selected from the 2011 World Series finals were rated by the participants. Each participant rated a randomly selected subset of 25 videos on the following emotional displays: happiness, sadness, somberness, confusion, anger, fear, concentration, confidence, excitement, aggression, hope, despair, and stress. Excitement was not considered in the analyses because during data collection, we found that participants interpreted the Dutch translation of excitement (*opgewonden*) in a sexual way, which is not what we intended to measure.

To determine how many emotion clusters would be needed to represent the emotion display ratings, we initially inspected 3-, 4-, and 5-factor solutions for a factor analysis on all 12 emotion ratings (with oblimin rotation) and found that each item loaded substantially (>0.50) on at least one of the factors in a 5-factor solution (which accounted for 64.4% of the total variance). Happiness loaded on a separate factor, without any substantial cross-loadings, and was therefore treated as a single-item scale for happiness. Anger and aggression loaded substantially on another factor, and also had few cross-loadings. They were treated as a two-item scale for anger (mean *r* = 0.65, range 0.37–0.82). The remaining three factors were difficult to interpret, had inter-correlations up to *r* = 0.63, and several items loaded on more than one of these factors. We therefore attempted a second exploratory factor analysis on the remaining items, which revealed no meaningful factor structure when asking for 4, 3, or 2 factors. We therefore constructed a third scale using all items with absolute factor loadings >0.50 on a one-factor solution (confidence [reverse-coded], sadness, somberness, confusion, fear, despair, stress) that we interpreted as worry (mean Cronbach’s α = 0.84, range 0.78–0.92).

The perceived emotion display ratings showed substantial inter-rater reliability^3^; happiness: ICC(2) = 0.81, 95% CI = [0.73, 0.87]; anger: ICC(2) = 0.88, 95% CI = [0.83, 0.92]; worry: ICC(2) = 0.88, 95% CI = [0.83, 0.92]. We also found moderate to strong inter-rater agreement; happiness: mean *r*_WG_ = 0.63, range 0.32–0.88; anger: mean *r*_WG(2)_ = 0.65, range 0.22–0.84; worry: mean *r*_WG(7)_ = 0.82, range 0.67–0.92. Less than moderate agreement (*r*_WG(J)_<0.51) was observed on 23 of the 189 ratings (12.2%), and each clip had moderate or better agreement on at least two of the three perceived emotion displays. As explained above, we deemed satisfactory inter-rater reliability (which we observed) more important than high inter-rater agreement because we focused on relative differences between clips, rather than assigning absolute perceived emotion display scores to the clips. Yet, we took inter-rater agreement into account as well by selecting 17 clips that offered a reasonable compromise between inter-rater agreement and variance in terms of perceived emotion displays. The characteristics of the clips we selected are displayed in **Table 1**.

**Table 1:** Means and associated ranges of *r*_wg_ values for each emotion cluster.

Season	Statistic	Happiness	Anger	Worry
2011 (*n* = 17)	*M*	2.39 (1.62–3.50)	3.27 (2.33–5.55)	3.91 (3.19–4.64)
	*r*_wg_ *σ*_E_ = 8.25	0.68 (0.39–0.88)	0.66 (0.22–0.84)	0.82 (0.72–0.91)
	*r*_wg_ σ_E_ = 5.09	0.47 (0.00–0.80)	0.35 (0.00–0.71)	0.32 (0.00–0.79)
2012 (*n* = 13)	*M*	3.11 (2.10–4.53)	3.11 (2.25–4.60)	3.53 (2.36–4.73)
	*r*_wg_ σ_E_ = 8.25	0.80 (0.66–0.91)	0.74 (0.40–0.90)	0.79 (0.63–0.91)
	*r*_wg_ σ_E_ = 5.09	0.44 (0.00–0.84)	0.44 (0.00–0.82)	0.35 (0.00–0.82)

*Ranges for Mean and r_WG(J)_ values are reported in brackets. In addition to the r_wg_ values based on the commonly used uniform null distribution (σ_E_ = 8.25) that we report in the text, we also report an alternative set of values for reference purposes, thereby following recommendations by LeBreton and Senter (2008). These alternative r_WG(J)_ are based on a moderately skewed null distribution (σ_E_ = 5.09).*

#### 2012 Finals Clips

All 29 clips selected from the 2012 World Series finals were rated by the participants. Because anger, happiness, and worry emerged as the most important emotion clusters in the 2011 finals, we adjusted the selection of rated emotion so that all items were relevant to one of these three emotions. The emotions assessed included: happiness, sadness, irritation, confusion, anger, worry, contentment, relaxed, cheerful, aggression, hope, despair, and stress.

Aggregation of the perceived emotion ratings proceeded in the same way as before. First, we checked the factor structure of the emotion ratings. After dropping hope, which did not clearly load on one of the factors, the expected three-factor solution emerged. Then, participant-level emotion scales were formed based on the individual perceived emotion display ratings. Happiness, contentment, relaxed, and cheerful formed the happiness scale (mean Cronbach’s α = 0.86, range 0.75–0.93); irritation, anger, and aggression formed the anger scale (mean Cronbach’s α = 0.81, range 0.68–0.89); and sadness, confusion, worry, despair, and stress formed the worry scale (mean Cronbach’s α = 0.81, range 0.68–0.88).

Once again, there was substantial inter-rater reliability^4^: happiness: ICC(2) = 0.92, 95% CI = [0.85, 0.95]; anger: ICC(2) = 0.95, 95% CI = [0.90, 0.97]; worry: ICC(2) = 0.91, 95% CI = [0.84, 0.95]. Inter-rater agreement again varied between modest and strong; happiness mean *r*_WG(4)_ = 0.82, range 0.66–0.91; anger mean *r*_WG(3)_ = 0.69, range 0.37–0.90; worry mean *r*_WG(5)_ = 0.78, range 0.63–0.91. We found less than moderate agreement (*r*_WG(J)_ <0.51) on only 6 of the 87 ratings (6.9%), and all clips showed moderate or better inter-rater agreement on at least two of the perceived emotion display ratings. The anger, happiness, and worry scales were therefore aggregated to the video level by averaging. From this set, we selected 13 clips that offered the best compromise between inter-rater agreement and variance in the perceived emotional displays for the main study. The characteristics of the clips we selected are displayed in **Table 1**.

## Discussion

This pre-study demonstrates that observers show considerable convergence in terms of the emotions they perceived in the facial expressions of pitchers. This is an important step to address our research question, but also indicates that emotional displays can be perceived even in short clips that include movements, where faces are partially covered by a baseball cap, and many times with facial hair as well. Thus, despite those sub-optimal circumstances individuals are able to recognize emotional expressions with considerable levels of inter-observer reliability.

The converging identification of emotions in the clips allowed us to categorize the clips according to the perceived emotions identified in them and to address our main research question concerning what information observers draw from those perceived emotions.

## Main Study

### Method

Thirty-four Dutch psychology undergraduate students (5 men, 29 women, age *M* = 20.00, range 19–26) viewed the 30 selected clips in random order. After viewing each clip, they first estimated the pitch speed (in km/h) and then rated the following anticipated outcomes on 7-point Likert scales (*1 = very unlikely*, *7 = very likely*): pitch speed (“The pitch will be fast” and “The pitcher will throw a slow pitch” (reverse-scored); mean *r* = 0.82, range 0.65–0.92); pitch difficulty (“The pitcher will throw a difficult ball” and “The pitcher will throw an easy ball” (reverse-scored); mean *r* = 0.80, range 0.47–0.95); pitch accuracy (“The pitch will be in the strike zone” and “The pitch will not be in the strike zone” (reverse-scored); mean *r* = 0.82, range 0.66–0.97); and batter swinging versus not swinging (“The batter will attempt to hit the ball” and “The batter will let the ball go” (reverse-scored); mean *r* = 0.77, range 0.53–0.96). The two items for each outcome were averaged. The study was carried out in accordance with APA regulations and approved by the IRB at the University of Amsterdam.

To be able to test whether the identified emotional displays were associated with objective game outcomes as well as to compare the predictions made by our participants with what actually happened during the games, we coded the outcome of each pitch as well as different aspects of the game that could influence the pitch or the decision to swing (or not swing) at the ball. All this information is publicly available from the television footage of the game as well as from several websites that provide statistical information about baseball matches. In addition to the game data, we obtained data regarding the pitchers’ and batters’ skills as recorded and reported on the MLB website (MLB.com)

The outcomes we coded were (1) the outcome of the pitch (hit/ball/strike/foul), (2) whether the batter swung or not, and (3) the speed of the pitch. We also recorded information about several contextual factors that we thought might impact the pitch or the decision to swing at the ball or not so that we could control for them in the analyses. Specifically, we recorded (a) whether the batter’s team was ahead or behind, (b) what inning it was, (c) whether the pitcher had already thrown two strikes or not, (d) whether the pitcher had already thrown three balls or not, (e) whether there were already two outs or not, and (f) the season batting average (taken from mlb.com) of the batter and (g) the pitching average for the pitcher (taken from mlb.com) as a measure of their skill (Appendix 1 contains an explanation of these baseball specific terms).^5^

### Results

Our analyses were conducted in three steps. In the first step, we assessed to what extent participants’ predictions of pitch outcomes were influenced by the perceived emotional displays in the clip. In the second step, we analyzed the accuracy of these predictions by comparing them to the actual outcomes of the pitch during the game. In the third step, we compared the relationship between the emotions detected to actual specific game outcomes.

#### Predicted Outcomes

The relation between the pitcher’s perceived emotional displays and the participants’ predictions was modeled by fitting GLMMs using the lme4 package (Bates et al., 2015) for R (R Core Team, 2015). Modeling started by fitting a full model that included fixed effects for one of the emotion clusters (i.e., happiness, anger, and worry), a random intercept for clip, and both a random intercept and random emotion slope for participants. Thus, three full models were fit for each prediction: one for happiness, one for anger, and one for worry. These models were then simplified to the reported models by dropping non-significant predictors one-by-one. The significance of the final predictors was tested using the “bootMer” method in the lme4 package (10,000 resamples) and reported using 98.3% confidence intervals (based on percentiles) to correct for fitting three models (significance level of α = 0.05/3 = 0.0167 – Bonferroni correction).

##### Pitch speed

Using the GLMM described above, we first regressed estimates of pitch speed in km/h on pitchers’ perceived emotional displays. We found that participants expected the pitch to be faster to the degree that the pitcher was perceived as expressing more anger (β = 0.24; 98.3% CI [0.06, 0.42]). Perceived expressions of happiness (β = -0.00; 98.3% CI [-0.26, 0.26]) and worry (β = -0.18; 98.3% CI [-0.38, 0.03]) did not influence estimated pitch speed. Repeating the analyses of the pitch speed prediction on the Likert scales revealed the same pattern. Again, the final models showed that participants expected the pitch to be faster when the pitcher was perceived as expressing more anger (β = 0.21; 98.3% CI [0.04, 0.39]), while perceived expressions of happiness (β = 0.07; 98.3% CI [-0.12, 0.27]), and worry (β = -0.16; 95% CI [-0.37, 0.04]) did not affect pitch speed estimates.

##### Pitch difficulty

The next set of analyses focused on estimates of pitch difficulty. We found that perceived displays of anger increased estimates of pitch difficulty (β = 0.15; 98.3% CI [0.03, 0.28]). Perceived happiness (β = 0.05; 98.3% CI [-0.09, 0.18]) and worry displays (β = -0.11; 98.3% CI [-0.25, 0.04]) did not influence estimates of pitch difficulty.

##### Pitch accuracy

Analyses of the predicted pitch accuracy showed that it was increased when the pitcher was perceived as expressing more happiness (β = 0.11; 98.3% CI [0.04, 0.18]) and decreased when the pitcher was perceived as displaying more worry (β = -0.11; 98.3% CI [-0.18, -0.03]). Perceived anger displays did not influence pitch accuracy estimates (β = -0.01; 98.3% CI [-0.10, 0.09]).

##### Batter swing

The final analyses focused on the prediction of whether the batter would swing or not. The final models indicated that participants were more likely to predict that the batter would swing to the degree that the pitcher was perceived as displaying more happiness (β = 0.10; 98.3% CI [0.03, 0.17]) or less worry (β = -0.09; 98.3% CI [-0.16, -0.02]). Perception of anger displays were not found to influence estimates of swinging (β = 0.00; 98.3% CI [-0.14, 0.13]).

##### Prediction results overview

Results regarding predictions of pitch quality indicate that (1) when pitchers’ were perceived as expressing anger estimated pitch speed and pitch difficulty increased, (2) perceived expressions of happiness increased estimated pitch accuracy, and (3) perceived expressions of worry decreased estimated pitch accuracy. With regard to predictions of the batter’s behavior, the batter was predicted to be more likely to swing at the ball to the degree that the pitcher was perceived as happy or less worried, a pattern that is consistent with that observed for pitch accuracy.

#### Accuracy of Predictions

Now that we have established that observers may use the perceived emotional expressions of pitchers to inform their predictions regarding pitch quality, the next question we wanted to address is whether perceived emotional expressions also predicted actual outcomes during the games. Since all of the clips were taken from actual matches during which several pitch characteristics were recorded, we could obtain information about the actual outcome of each pitch. We could then compare the predictions made by our participants with what occurred in reality. One aspect that we could not assess using the recorded data on the actual game outcome is pitch difficulty. This subjective information is not recorded. However, one can assume that faster pitches are on average harder to hit, and therefore we tested whether the prediction of pitch difficulty was related to speed. The other aspects that are objective – pitch speed, pitch accuracy (as determined by the umpire – the baseball referee), and swing or not swing were all used in the analysis.

Because the actual pitch outcomes are on the clip level of analysis, we first needed to aggregate participants’ predictions of pitch outcomes to the clip level. Three predictions had high inter-rater reliability: pitch speed (in km/h): ICC(2) = 0.89, 95% CI = [0.81, 0.93]; pitch speed (Likert scale): ICC(2) = 0.88, 95% CI = [0.78, 0.92]; and pitch difficulty: ICC(2) = 0.71, 95% CI = [0.48, 0.82]. The inter-rater reliability was not significant for pitch accuracy (ICC(2) = 0.35, 95% CI = [0.00, 0.59]) and swinging versus not swinging (ICC(2) = 0.31, 95% CI = [0.00, 0.56]), as indicated by the confidence intervals that both include 0 (which is the lower bound for ICC(2) values). The proportion of resamples equaling 0 was less than 5% in both cases (3.5 and 4.4%, respectively), which constitutes marginally significant evidence that the differences between these predictions may still be (partially) attributed to differences between clips. We therefore decided to proceed with aggregating all predictions to the clip level by averaging, while noting that the predictions of swinging and accuracy are less reliable than the other two predictions.

#### Relation Between Predictions and Actual Outcomes

To assess the accuracy of the participants’ predictions, we compared these predictions to three actual pitch outcomes that we recorded for each clip (pitch speed, hit/ball/strike/foul, and batter swinging vs. not swinging). Each prediction was separately used to predict these outcomes. In these analyses, we included all coded game factors (batter’s team leading or behind, two outs or not, etc.) in order to control for any influence that these game factors may have on the pitcher’s and/or batter’s behavior. Because including all seven game factors in the analysis substantially reduced the remaining degrees of freedom (based on 30 clips in total), thereby reducing our statistical power, we also repeated each analysis without controlling for game factors. The reported statistics control for game factors, but in cases where these effects approach significance, we also reported the analysis without controlling for game factors to increase statistical power.

##### Pitch speed

The first set of analyses focused on the actual pitch speed. Actual pitch speed was normally distributed (Shapiro–Wilk’s *W* = 0.96, *p* = 0.311). We separately regressed the actual pitch speed on the estimated pitch speed in km/h, estimated pitch speed on the Likert scale, and estimated pitch difficulty. None of these estimates was related to the actual pitch speed: pitch speed in km/h: β = -0.09, *t* = -0.33, *p* = 0.747; pitch speed on a Likert scale: β = -0.01, *t* = -0.03, *p* = 0.976; and pitch difficulty: β = -0.24, *t* = -0.98, *p* = 0.337.

##### Pitch accuracy

The second set of analyses focused on the accuracy of the pitch. For this purpose, we recoded pitches that resulted in ‘ball’ as inaccurate pitches (*N* = 10) and the remaining pitches as accurate pitches (for 1 pitch the outcome was unclear, and this pitch was not included in the analysis). A logistic regression was used to account for this dichotomous coding. The relation between estimated pitch accuracy and the actual pitch being in the strike zone did not reach statistical significance when controlling for game factors (*OR* = 2.41, Wald’s *z* = 1.52, *p* = 0.129), but was marginally significant when game factors were not controlled for (*OR* = 2.14, Wald’s *z* = 1.72, *p* = 0.085). Thus, there was some suggestive evidence that whether the pitch would end up in the strike zone could be predicted with a certain degree of accuracy from viewing a video clip of the pitcher.

##### Batter swing

In a logistic regression, we regressed actual swinging versus not swinging on the prediction of swinging made by our participants. The initial analysis revealed no relation between predicted and actual swinging (*OR* = 2.50, Wald’s *z* = 1.32, *p* = 0.188), but when game factors were not controlled for this relationship became significant, *OR* = 2.43, Wald’s *z* = 2.03, *p* = 0.042. Thus, we found evidence that a batter’s behavior in terms of swinging or not swinging at a ball can be predicted with a reasonable degree of accuracy based on a short video clip of the pitcher just before his throw.

#### Relation Between Perceived Emotion Displays and Actual Outcomes

Next we examined whether the perceived emotional displays of the pitchers predicted objective qualities of their pitches. We regressed each of the three actual outcomes (pitch speed, pitch in strike zone [i.e., not ‘ball’], and batter swinging vs. not swinging) in the selected clips on the pitchers’ perceived emotional displays, as identified in the pre-study. As above, the reported statistics reflect the relation between perceived emotion displays and game outcomes while controlling for all coded game factors.

##### Pitch speed

A first series of analyses showed that the pitcher’s perceived emotion displays were unrelated to the speed of the actual pitch: happiness: β = 0.31, *t* = 1.48, *p* = 0.156; anger: β = -0.28, *t* = -1.15, *p* = 0.262; worry: β = 0.05, *t* = 0.22, *p* = 0.831. Thus, actual pitch speed was neither related to the participants’ predictions, nor to the pitchers’ perceived emotional displays.

##### Pitch accuracy

The second series of analyses showed that the actual accuracy of the pitch (i.e., whether it was not a ‘ball’) was also unrelated to the pitcher’s perceived emotional displays happiness: *OR* = 1.39, Wald’s *z* = 0.63, *p* = 0.532; anger: *OR* = 0.21, Wald’s *z* = -1.23, *p* = 0.218; worry: *OR* = 1.13, Wald’s *z* = 0.26, *p* = 0.793.

##### Batter swing

Regarding the relation between the pitcher’s perceived emotional displays and the batter’s swinging, perceived displays of anger (*OR* = 0.68, Wald’s *z* = -0.59, *p* = 0.558), worry (*OR* = 0.81, Wald’s *z* = -0.41, *p* = 0.682) and happiness (*OR* = 2.23, Wald’s *z* = 1.37, *p* = 0.172) did not predict batters’ actual swinging.

##### Pitcher’s perceived happiness displays and prediction of actual outcomes

Our final analysis focused on the relation between the pitchers’ perceived happiness, predictions about swinging, and actual swinging. The pitcher’s perceived displays of happiness were unique in the sense that they influenced predicted swinging, which in turn was related to actual swinging. In addition, the pitcher’s displays of happiness had a comparatively stronger relation to actual swinging than the other perceived emotional displays. We therefore wondered whether our participants’ predictions regarding the batters’ swinging could explain part of the covariance between the pitchers’ perceived displays of happiness and the batters’ actual swinging behavior. We tested this possibility by conducting a mediation analysis. It should be noted that this was not a mediation analysis in the traditional sense because we were not testing a process (i.e., it is impossible that batters were actually swinging *because* our participants were estimating their swinging based on the perceived pitchers’ expressions). We merely tested whether the batter’s actual swinging is related to the variance that is shared between perceptions of happy expressions and predictions of swinging.

We determined and bootstrapped (25,000 resamples) the indirect effect of perceived happiness on actual swinging through predicted swinging. Two hundred and thirty resamples (0.9%) that were based on models that could either not be fit (e.g., because of a lack of variance in the DV), or that produced extreme outliers in the distribution of coefficients in the form of very large coefficients (we used 4 as a cutoff value, which translates into an Odds Ratio of over 50) were dropped. Confidence intervals based on the remaining resamples that exclude *OR* = 1.000 indicate significance.

The results indicated that the indirect effect of perceived happiness through predicted swinging on actual swinging was not significant at the conventional α = 0.05 level, *OR* = 1.44, 95% percentile-based CI: [0.926, 3.164]. The 90% percentile-based CI [1.003, 2.643] indicated that the effect was significant at the α = 0.10 level, which constitutes marginally significant evidence that the batter’s actual swinging was indeed related to the variance shared by the perceived pitcher’s expressions of happiness and participants’ predictions about swinging.

## General Discussion

The idea that displays of emotions provide information to others is well established (e.g., Parkinson, 1996; Keltner and Haidt, 1999; Manstead and Fischer, 2001; Van Kleef, 2009). It is also clear from previous research that emotions can have a pervasive impact on sports performance (e.g., Totterdell, 2000). Building on and extending pervious work, the current research shows that perceived displays of emotion may be used to predict future behavior and actions of players in sports games. Answering a recent call by Friesen et al. (2013), we examined the interpersonal dynamics of emotions in the context of professional competitive sports. Specifically, we used archival data of the MLB finals to investigate (1) whether observers can reliably perceive players’ emotional displays, (2) what types of information observers distill from these perceived emotional displays, and (3) whether observers’ predictions regarding the players’ future behaviors based on their perceived emotional expressions matched actual behaviors exhibited by the players.

The results allow for three broad conclusions. First of all, our data indicate that several emotions can be reliably identified during professional sports games, even when the displays are short (only a few seconds) and often partly obscured by baseball caps, shadows, facial hair, etc. Specifically, we found consistent evidence across two data sets that displays of happiness, anger, and worry were reliably perceived by observers.

Second, we found evidence that observers used pitchers’ perceived emotional displays as information when attempting to assess future pitch features and behaviors of batters (swinging vs. not swinging) who were facing the pitcher who displayed the emotion. To the degree that participants perceived a pitcher as displaying more anger, they predicted his future pitch to be faster and more accurate. To the degree that participants perceived a pitcher as displaying more worry, they predicted his future pitch to be slower and less accurate. And to the degree that participants perceived a pitcher as displaying more happiness, they predicted that the future pitch would be more accurate, and that the batter would be more likely to swing at the ball.

Third, across the board, participants’ predictions regarding pitch speed, accuracy, and difficulty did not converge well with actual pitch qualities. However, we did find some evidence that participants’ predictions regarding the batter’s swinging behavior based on the pitcher’s expressions of happiness were associated with the batter’s actual swinging. Even though this effect was only marginally significant, and although there was only modest inter-rater agreement about the prediction of swinging, we believe that this effect is potentially important and as it pertains to a key aspect of the game – attempting to hit the ball – attests to the interpersonal power of emotions in natural settings.

### Theoretical and Practical Implications

Research on the social effects of emotions has thus far mostly used emotional displays that have been manipulated either by a trained confederate (e.g., Barsade, 2002; Cheshin et al., 2011), verbal text messages (e.g., Van Kleef et al., 2004), selected photos or videos (e.g., Tiedens, 2001; Kopelman et al., 2006; Van Doorn et al., 2015b), emotion induction (Sy et al., 2005), instructions to show emotions (e.g., Sinaceur and Tiedens, 2006; Heerdink et al., 2013), or assessment of emotional displays that are required by one’s job (e.g., Barger and Grandey, 2006). In contrast, our study examined a setting in which emotions occurred naturally. Although a few earlier studies have investigated the social effects of emotions as they naturally occur in (sports) teams (e.g., Totterdell et al., 1998; Totterdell, 2000), this research has been limited to the affective reactions that may be triggered by emotional expressions. To the best of our knowledge, the current study is the first to consider the inferences people draw from the perception of others’ emotional displays in settings that are not staged and where emotional displays are not prescribed by the job (cf. ‘service with a smile’ in customer service). Pitchers in baseball are not required to display specific emotions, and if anything they might be trying to disguise their emotions. Our study thus sheds initial light on the inferences observers may draw from naturally occurring perceptions of emotional displays in the context of actual, high-stakes interactions.

It has been suggested that encountering emotional displays of others helps individuals to make sense of ambiguous situations and to predict the behavior of other individuals in the social situation (Van Kleef et al., 2010, 2011). To date, however, most research on the social effects of emotions has tended to focus on concurrent effects. That is, previous work has focused on how people use social-emotional cues to inform their understanding of past or ongoing events (e.g., Knutson, 1996; Van Kleef et al., 2004; Hareli et al., 2009; Van Doorn et al., 2012, 2015a). The current work advances this field of inquiry by showing that perceived emotional displays may also be used to inform predictions about future behavior.

Participants in our study did not receive specific instructions to focus on the emotions displayed by the pitcher or to use them as cues. Yet, it is evident that they did so, because the perception of the pitchers’ emotional displays predicted participants’ judgments of the pitchers’ throw. This finding supports the theoretical notion that when trying to predict other people’s behavior, individuals use cues from others’ emotional displays to inform their judgments (Van Kleef et al., 2011). Specifically, our findings indicate that individuals use others’ perceived emotional displays to make predictions regarding the future behavior of the expressers (in the current study, the pitchers) as well as the future behavior of observers (the batters).

### Limitations, Strengths, and Future Directions

Even though our results allow for a number of clear conclusions, some patterns in the data are inconclusive. For instance, the absence of evidence for a relation between perceived emotional expressions and actual pitch outcomes may reflect that these relations are simply not there, but could also indicate that these relations were too inconsistent to detect in the current study. Indeed, it is likely that in a real game, especially such loaded games as the World Series finals, many different factors influence the various pitch outcomes that we analyzed, which may overshadow or limit any emotional influences. One reason why the effects of pitchers’ perceived emotional displays on batters’ behavior are not stronger might be due to the inherent time pressure and the limited cognitive resources available to the batters. EASI theory holds that the influence of inferential processes in response to emotional expressions is reduced when perceivers’ information processing motivation and/or ability are reduced (Van Kleef, 2009), and a growing body of research supports this idea (for reviews, see Van Kleef et al., 2011, 2012). The amount of time batters have between the time they see the emotion of the pitcher and the time that the pitch reaches them and they need to decide how to respond is very short – no more than 3 s. Because of these pressures the amount of information that batters are motivated and able to distil from the perception of the pitchers’ emotional displays might be reduced. Future research could investigate this possibility more directly by examining a sports setting that allows for greater variability in the time lag between one player’s emotional expression and another player’s response (e.g., chess).

During a baseball match, players are confronted with a lot more information than just the facial displays of their counterparts. It is possible, therefore, that players during a match would focus less on the emotions of the pitcher than our participants did. Our findings may therefore represent an overestimation of actual effects as they take place during competitive sports games. Yet, one can argue that players in competitive sports games could potentially glean useful information from their counterpart’s emotional expressions if they were to pay close attention to them.

It is important to note that we have investigated the observable, perceived displayed emotion that was communicated (intentionally or not) and not the actual feelings of the players. It is possible that these displays were inauthentic and were purposely displayed as part of one’s role (Rafaeli and Sutton, 1987) in order to try and influence the opponent. It is also possible that what observers saw were not displays of emotions at all, but rather physical strain of the players who were engaging in a physically strenuous activity. Regardless of what the displayers were actually feeling, however, these displays could reliably be interpreted as emotional expressions, and did lead to inferences and predictions regarding future actions.

We found that pitchers were assessed as expressing relatively little emotion in the baseball clips we used, and this limited variance may have reduced the magnitude of some of our effects. The highest-intensity perceived emotional displays were around the midpoint of the scale (5.5 out of 10), and many displays were considerably lower in intensity. One reason for the limited amount of emotion displays detected might be the fact that the expressers are professional sports players who perform at the highest level and who are able to control their emotions and might in fact receive training on how to do so (suggested in Friesen et al., 2013). Baseball caps, facial hair and placing the glove in front the face before the pitch are all strategies that might be used on purpose as a means to disguise or blur the emotions the pitcher is feeling or displaying. The relatively low intensity displays identified by our participants might indicate that professional baseball players know that emotions carry information, which may lead them to try and hide or down-regulate their emotions in the same way as professional poker players do. Despite all these confines our participants were able to consistently detect cues of anger, happiness, and worry on pitchers’ faces. Thus, even when naturally occurring and relatively mild emotional expressions are not related to objective pitch outcomes, deliberate and more intense emotional expressions of the pitcher could still influence the batter.

Another limitation of the current study is the fact that our participants viewed the pitcher on a computer screen taken from TV footage. This is different from what the actual batters saw, as they viewed the pitcher face-to-face from a distance of about 18 meters. Thus, the size of the face and the body were not in the same proportion between the two types of observers. Our participants viewed larger footage of the pitchers that filled most of the computer screen, whereas the batter had less of a “zoom in” option and the pitcher was smaller in proportion to all the other things in their field of view. Yet, one should take into account the fact that the pitcher communicates with the catcher who stands behind the pitcher (and is therefore even farther away; see **Figure 1**), with the help of his fingers. Accordingly, if a pitcher can clearly see the fingers of the catcher, it is safe to assume that the batter can see the face of the pitcher sufficiently well to notice nuances such as facial displays of emotion, in addition to body movements. Moreover, professional baseball players, as evident by the quote mentioned by Charlie Metro, attest to not only noticing facial expressions of pitchers but also to using them as cues.

As outlined in EASI theory (Van Kleef, 2009), emotional expressions can influence observers’ behavior by eliciting inferential processes and/or affective reactions in them (see also Hareli and Rafaeli, 2008). Thus, observing the emotions of the pitcher might influence the batter not only by providing him with information regarding the pitch, but also by influencing his own emotion. These elicited emotions could also influence the batter’s actions. Because we have no information regarding the batters’ emotional experiences, we cannot rule out the possibility that the batters’ emotional responses to the pitchers’ perceived emotional displays impacted on their decisions to swing or not swing. Future studies should therefore incorporate both affective and inferential processes so that their relative impact can be disentangled (cf. Van Kleef et al., 2009).

Perhaps the biggest limitation of the study is the fact that we could not use all the possible data points, that is, all the pitches that were thrown. Our data relied on TV footage, which showed the pitcher’s face only in about 10% of the cases. In most instances the batter was the one who was being captured on camera, as well as players on bases, the coaches, or the crowd. Even though we have no reason to assume a systematic bias on the part of the producers of the TV footage to show some pitchers and not others, such a bias cannot be ruled out on the basis of the available data.

## Conclusion

These limitations notwithstanding, the present study attests to the pervasive power of emotional expressions in sports. Our findings indicate that observers use the perceived emotional displays of professional pitchers during baseball games to arrive at predictions regarding objective qualities of the pitch as well as behavioral responses of the batter. This conclusion suggests that professional sports performance is influenced by emotional expressions and implies that performance can potentially be improved by taking this into account. Being able to identify and unravel the information that is conveyed by emotional displays could very well lead to a “home run”.

## Author Contributions

AC and GvK developed the study idea and design, AC and JK conducted the data arrangement and collection, and MH, AC and GvK conducted the analyses. All authors were involved in writing the report and approved the current version.

## Conflict of Interest Statement

The authors declare that the research was conducted in the absence of any commercial or financial relationships that could be construed as a potential conflict of interest.

## Footnotes

1 This included a clear shot of the face of the pitcher right before the pitch, when the pitcher’s face was present in the shot for more than 1.5 s, and if the pitch itself was thrown no more than 2 s after the pitcher was depicted. Clips that matched the above mentioned criteria were further screened to ensure that there were clips of every pitcher, with at least two possible outcomes (i.e., swing, ball, foul, or strike). After selecting the pitchers and pitches that were usable for the experiment, videos were included in the study if there were four clips of the same pitcher. This was done to ensure some variance in pitchers’ perceived emotional displays and to take into account specific features of a pitcher (not their momentary emotional display) that had an impact on predictions.

2 We contacted the US Major League Baseball Association with a request for footage from their cameras that were directed at the pitcher. To this day no response was received.

3 The emotion ratings were not normally distributed. Because we are not aware of a method to correct the ICC for non-normality of the DV, and because simulation studies show that non-normality does not inflate the chance of Type I errors with the *F*-test on which the ICC is based (e.g., Lix et al., 1996), we report ICC values based on parametric tests. Given the high ICC values that we obtained, we consider it unlikely that a non-parametric test would lead to opposite conclusions about the reliability of the ratings.

4 The emotion ratings were not normally distributed. See comment for the 2011 series.

5 We also coded whether third base was occupied or not during the clip, but there were only three clips in total, all from the 2011 finals, in which third base was occupied. There was therefore too little variance to include this as a covariate.
